# Cardiac autonomic function in patients with acute exacerbation of chronic obstructive pulmonary disease with and without ventricular tachycardia

**DOI:** 10.1186/s12890-016-0287-0

**Published:** 2016-08-20

**Authors:** Xingde Wang, Zhaohua Jiang, Bin Chen, Li Zhou, Zhibin Kong, Sheng Zuo, Hua Liu, Shaojun Yin

**Affiliations:** 1 Department of Cardiology, Shanghai Jiao Tong University Affiliated Sixth People’s, Hospital, Shanghai, 200233 China; 2 Department of respiratory medicine, Shanghai Pu Nan Hospital, Shanghai, 200125 China; 3 Department of respiratory medicine, Shanghai Jiao Tong University Affiliated Sixth, People’s Hospital, Shanghai, 200233 China

**Keywords:** Chronic obstructive pulmonary disease, Acute exacerbation, Electrocardiogram, Autonomic nervous system, Ventricular tachycardia, Phase-rectified signal averaging (PRSA)

## Abstract

**Background:**

Autonomic dysfunction in patients with chronic obstructive pulmonary disease (COPD) may increase the risks of arrhythmia and sudden death. We studied cardiac autonomic function in patients with acute exacerbation of COPD (AECOPD).

**Methods:**

Patients with AECOPD were classified into ventricular tachycardia (VT) and non-VT groups according to the presence or absence of VT. The following parameters derived from 24-h Holter monitoring were compared between groups: average heart rate, heart rate deceleration capacity (DC), heart rate acceleration capacity (AC), standard deviation of normal RR intervals (SDNN), standard deviation of average RR interval in 5-min segments (SDANN), root mean square of standard deviations of differences between adjacent normal RR intervals (rMSSD), low-frequency power (LF), high-frequency power (HF) and LF/HF ratio.

**Results:**

Seventy patients were included, 22 in the VT group and 48 in the non-VT group. The groups had similar clinical characteristics (except for more common amiodarone use in the VT group, *P* < 0.05) and general ECG characteristics. DC, SDNN, SDANN and rMSSD were lower and AC higher in the VT group (*P* < 0.05). In the VT group, DC was correlated positively with SDNN (*r* = 0.716), SDANN (*r* = 0.595), rMSSD (*r* = 0.571) and HF (*r* = 0.486), and negatively with LF (*r* = -0.518) and LF/HF (*r* = -0.458) (*P* < 0.05). AC was correlated negatively with SDNN (*r* = -0.682), SDANN (*r* = -0.567) and rMSSD (*r* = -0.548) (*P* < 0.05).

**Conclusions:**

DC decreased and AC increased in patients with AECOPD and VT, reflecting an imbalance in autonomic regulation of the heart that might increase the risk of sudden death.

## Background

Chronic obstructive pulmonary disease (COPD) is a common, serious lung disease that impacts severely on patient quality of life. COPD inflicts a heavy financial burden on patients, their families and society, and its incidence and mortality rate have gradually increased in recent years [1]. In the USA, the mortality and incidence of COPD rank third and second, respectively, of all diseases [2]. COPD incidence in China is 8.2 % in those older than 40 years, and the disease ranks fourth and third among causes of death in urban and rural areas, respectively [3]. Cardiovascular diseaseis the leading cause of death in mild to moderate COPD, but those with severe disease more common die of respiratory failure, and in some cases mortality is associated with the occurrence of arrhythmia [4, 5]. About 31.9 % of patients with COPD suffer from ≥ 30 premature ventricular beats per hour, and the incidences of non-sustained and sustained ventricular tachycardia (VT) are 13.0 % and 1.6 %, respectively [6]. Furthermore, COPD and its severity correlates independently with non-sustained VT [6].

The pathogenesis of COPD is not fully understood. Inhalation of deleterious particles/gases tends to induce pulmonary oxidative stress, an imbalance between protease and antiprotease function, and the generation of inflammatory mediators that recruit neutrophils, damage pulmonary structures and restrict airflow [7]. Autonomic nervous system (ANS) dysfunction is now recognized as playing an important role associated with COPD pathogenesis [7]. Sympathetic autonomic nerves act to quicken the heart and strengthen the heart rate acceleration capacity (AC). In contrast, the vagal nerve slows the heart rate and enhances the heart rate deceleration capacity (DC). An imbalance between these two arms of the ANS can promote the occurrence of lethal arrhythmia [8, 9]. An acute exacerbation of COPD (AECOPD) aggravates the airflow obstruction and imbalance in autonomic function, potentially resulting in malignant arrhythmia and sudden cardiac death [10–12].

Various non-invasive diagnostic techniques are commonly used to assess cardiac autonomic function in patients with COPD, including the Ewing test, heart rate variability (HRV), baroreflex sensitivity (BRS), heart rate turbulence (HRT), and cardiac chronotropic and QT interval analysis. The Ewing test is a cardiovascular reflex examination involving 5 items [13], but has certain limitations, such as the necessity for active cooperation of the patient and manual implementation, and a susceptibility to subjective factors. The other commonly used techniques for testing autonomic function also have limitations, as most rely on indirect methods such as detection of physiologic parameters regulated by autonomic nerves. For example, the indirect reflex regulation of blood pressure and heart rate by autonomic nerves is used as the basis for HRV and HRT measurements. It is also difficult to differentiate between the effects of sympathetic and vagal nerves since a change in the parameter measured reflects the combination of their influences, leading to qualitative rather than quantitative assessments in most cases. Moreover, it can be challenging to distinguish between the effects of physiologic and pathologic factors. An additional drawback of some techniques is that assessments can only be made under certain conditions. For example, HRT measurements can only be carried out after the appearance of ventricular premature beats, and BRS can only be determined by the injection of phenylephrine.

Measurements of DC and AC tend to avoid many of the limitations described above. DC and AC can quantitatively assess vagal and sympathetic nerve tone over a 24-h period, and screening of DC can provide an early warning to patients at high risk of sudden death [14]. The phase rectified signal average (PRSA) technique can extract periodic signals from a complicated time series consisting of periodic cardiac electrical signals contaminated with variable signal noise and artifacts. An advantage of this method is that no other treatments are needed, minimizing the number of factors that might affect the measurements, thereby improving specificity and sensitivity. The PRSA techniqueis thought to be superior to other traditional methods in the detection of quasi-periodic signals in long-duration heart rate records that represent the regulatory function of cardiac autonomic nerves. Thus, the data provided by this approach likely have high clinical relevance.

Several studies have reported that DC is significantly decreased in patients with acute myocardial infarction, diabetes mellitus and COPD, and can be used as an indicator of autonomic function and an early warning of sudden death [14–18]. Our previous study indicated both DC and AC of heart rate decreased in patients with hemispheric infarction,reflecting a decrease in both vagal and sympathetic modulation [19]. However, there are few data concerning the utility of DC as a gauge of cardiac autonomic function in patients with AECOPD. The aim of this study was to measure the changes in DC and AC in patients with AECOPD accompanied with VT, and investigate the role of these parameters in the evaluation of cardiac autonomic function by determining their correlations with other commonly used indicators.

## Methods

### Patients

This was a retrospective observational study of patients with AECOPD and arrhythmia admitted to the Sixth People’s Hospital affiliated with Shanghai Jiao Tong University (Shanghai, China) between March 2010 and March 2014. Seventy patients were included, and AECOPD was diagnosed based on the 2013 criteria of the Global Initiative for Chronic Obstructive Lung disease [20]. COPD exacerbations were defined as events of acute onset, characterized by a worsening of the patient’s respiratory symptoms (baseline dyspnea, cough, and/or sputum production) that is beyond normal day-to-day variations and leads to a change in medication. Arrhythmias were defined as isolated premature atrial beats, supraventricular tachycardias, isolated and paired premature ventricular complexes, and combined ventricular arrhythmias consisting in ventricular tachycardias. Details of the medical history, clinical examination and supplementary investigations were obstained from medical records, and patients were excluded from the analysis if any of the following exclusion criteria applied: hypertension; previous acute myocardial infarction; diabetes mellitus; severe liver or kidney dysfunction; tumors; anemia; drugs that might affect HRV (e.g. β-adrenoceptor antagonists); basal heart rate not in sinus rhythm (e.g., atrial fibrillation or flutter); and an implanted pacemaker. The study was approved by the ethics committee of the Sixth People’s Hospital affiliated with Shanghai Jiao Tong University. All patients provided informed written consent for their anonymized data, including electrocardiogram (ECG), to be analyzed and included in a subsequent research study.

### Study design

Patients were allocated into two groups: a VT group (VT present) or a non-VT group (VT absent). VT was defined as at least three consecutive ventricle-originated extrasystoles occurring at a frequency of > 100 beats/min in the dynamic ECG.VT was classified as non-sustained if it persisted for < 30 s, and sustained if it persisted for 30 s or more [21].

### Collection of clinical data

The following data were obstained from the medical records: patient gender; patient age; smoking exposure, and concurrent medications, including β_2_-adrenoceptor agonists, anticholinergics, calcium channel antagonists, amiodarone and hormone replacement therapy. Biochemical analyses, including blood urea nitrogen (BUN), serum creatinine (Cr), fasting blood glucose (FBG) and serum total cholesterol (TC), arterial blood gas analyses and electrolytes, were carried out on the day following admission. The Modified British Medical Research Council(mMRC) questionnaire on breathlessness was used to assess symptoms [22]. Echocardiographic measurement of left ventricular ejection fraction (LVEF) was performed within 1 week of admission. Dynamic electrocardiography was carried out within 1 week of admission to obtain measurements of minimum, maximum and average heart rates and assess the characteristics of premature ventricular beats (when present). Echocardiography and ECG investigations were performed by experienced physicians, and offline data analysis was performed by experienced clinicians, who were blind to the patient grouping. None of the enrolled patients underwent pulmonary function testing as part of this study.

### Measurement of DC and AC

All patients underwent 24-h Holter monitoring, and the data were analyzed offline using a DMS dynamic cardiograph analysis system (DM Software, NV, USA). Interference and artifacts were removed through the man-machine dialog, and DC and AC values were calculated automatically by the analysis system. The analysis was performed by a certified ECG technician supervised by a cardiovascular physician (both blind to the study hypothesis).

DC was measured as follows [14]: the deceleration period was screened from the 24-h dynamic ECG using a 4096 Hz sampling frequency digital automatic processing system, with the size of the heart rate segment was fixed at 20 cycles (i.e., RR intervals). The deceleration point of a prolonged cardiac cycle compared with the previous one was selected as the ‘anchor point’ for ordered arrangement of different heart rate segments, and mean values were calculated for the following parameters: X0, the mean of the RR intervals for all anchor points; X_1_, the mean of the RR intervals for all the cardiac cycles immediately following the anchor points; X_-1_, the mean of the RR intervals for all the cardiac cycles immediately preceding the anchor points; and X_-2_, the mean value of the RR intervals for all the cardiac cycles immediately preceding the X_-1_ points. The mean values of X_0_, X_1_, X_-1_ and X_-2_ were calculated separately, and the DC value calculated as: DC (ms) = (X_0_ + X_1_ – X_-1_ – X_-2_)/4.

The acceleration period was screened using similar methods, and the calculated parameters were substituted into the above formula to obtain the AC value, which was negative.

### Assessment of HRV

Each HRV index was calculated automatically using the DMS analysis software. The time-domain indexes (units, ms) included: SDNN (the standard deviation of all normal RR intervals), SDANN (the standard deviation of the average normal RR intervals in all 5-min segments) and rMSSD (the root mean square value of the standard deviations of the successive differences between adjacent normal RR intervals in the whole 24-h period). The frequency-domain indexes (units, ms2) included: LF (low-frequency power), HF (high-frequency power) and the LF/HF ratio.

### Statistical analysis

Statistical analyses were performed using SPSS16.0 software (SPSS Inc., Chicago, IL, USA). All measurement data were subjected to tests of normality. Normally distributed data are reported as the mean ± standard deviation. For normally distributed data, comparisons between groups were performed using Student’s t-test, and correlations between variables analyzed using Pearson correlation analysis. Data not normally distributed were analyzed with Spearman correlation analysis. Count data are expressed as number of cases and percentages, and comparisons between groups were performed using the χ2 test. *P* < 0.05 was considered statistically significant.

## Results

### Clinical characteristics of the patients

Seventy patients were included in the final analysis, 22 (aged 50–78 years) in the VT group and 48 (aged 51–79 years) in the non-VT group. The clinical characteristics of these patients are presented in Table 1. The proportion of patients taking amiodarone was significantly higher in the VT group than in the non-VT group (68.2 % vs. 10.4 %, *P* < 0.05). There were no significant differences between groups in the other clinical characteristics (Table 1).

**Table 1:** Clinical characteristics of the 70 patients included in the study

Variable	Non-VT group (*n* = 48)	VT group (*n* = 22)	P
Gender (male)	31 (64.5 %)	15 (68.2 %)	0.77
Age (years)	61.2 ± 11.4	60.6 ± 11.8	0.86
BUN (mmol/L)	6.0 ± 1.5	6.1 ± 1.2	0.84
Cr (μmol/L)	76.3 ± 15.8	77.1 ± 15.2	0.82
FBG (mmol/L)	5.0 ± 0.9	5.1 ± 0.8	0.79
TC (mmol/L)	4.8 ± 0.6	4.7 ± 0.7	0.83
LVEF (%)	59.8 ± 6.4	58.7 ± 6.5	0.87
β2 agonist use	45 (93.8 %)	20 (90.9 %)	1.00
Anticholinergic use	40 (83.3 %)	19 (86.4 %)	1.00
Hormone replacement therapy	36 (75.0 %)	17 (77.3 %)	0.84
Calcium antagonist use	15 (31.2 %)	10 (45.5 %)	0.25
Amiodarone use	5 (10.4 %)	15 (68.2 %)	<0.05
Smoker	10 (20.8 %)	6 (27.3 %)	0.64
PH	7.40 ± 0.05	7.41 ± 0.08	0.13
PaO2(mmHg)	60.5 ± 5.9	59.8 ± 5.3	0.73
PaCO2(mmHg)	49.4 ± 5.6	50.5 ± 5.9	0.43
Serum K+(mmol/L)	4.2 ± 0.4	4.1 ± 0.4	0.25
Serum Na + (mmol/L)	138.8 ± 3.3	139.7 ± 3.2	0.12
Serum Cl-(mmol/L)	99.8 ± 2.0	98.8 ± 2.2	0.06
mMRC Grade 0	10 (20.8 %)	5 (22.7 %)	0.88
mMRC Grade 1	15 (31.2 %)	6 (27.3 %)	0.80
mMRC Grade 2	12 (25 %)	5 (22.7 %)	0.87
mMRC Grade 3	8 (16.7 %)	4 (18.2 %)	0.89
mMRC Grade 4	3 (6.3 %)	2 (9.1 %)	0.69

Data presented as n (%) or mean ± standard deviation

*BUN* blood urea nitrogen, *Cr* serum creatinine, *FBG* fasting blood glucose, *LVEF* left ventricular ejection fraction, *TC* serum total cholesterol, *VT* ventricular tachycardia, *mMRC* modified British Medical Research Council questionnaire

### Comparison of general Holter recordings characteristics between the VT and non-VT groups

Of 22 patients with VT, 20 (90.9 %) had non-sustained VT and 2 (9.1 %) had sustained VT (Table 2). Therefore, non-sustained VT and sustained VT occurred in 28.6 % and 2.9 % of all patients with AECOPD in our study. There were no significant differences between the non-sustained VT and sustained VT groups in the general Holter recordings parameters assessed (average, minimal and maximal heart rate, mean ventricular premature beats, and proportion of patients with ≥ 30 ventricular premature complexes/hour; Table 2).

**Table 2:** Comparison of the general Holter recordings characteristics between patients in the VT and non-VT groups

Variable	Non-VT group (*n* = 48)	VT group (*n* = 22)	P
Average heart rate (beats/min)	79.5 ± 13.8	81.2 ± 15.8	0.83
Minimal heart rate (beats/min)	70.5 ± 13.6	73.8 ± 15.3	0.86
Maximal heart rate (beats/min)	90.1 ± 16.4	94.8 ± 17.2	0.86
Mean ventricular premature beats	1257.3 ± 251.7	1326.2 ± 268.5	0.83
Ventricular premature complexes ≥ 30/h	19 (39.6 %)	9 (40.9 %)	0.92
Non-sustained VT	0	20	
Sustained VT	0	2	

Data presented as *n* (%) or mean ± standard deviation

*VT* ventricular tachycardia

### Comparison of DC, AC and HRV parameters between the VT and non-VT groups

Patients in the VT group had a lower DC, SDNN, SDANN and rMSSD, and a higher AC, than patients in the non-VT group (all *P* < 0.05; Table 3). However, there were no significant differences between the VT and non-VT groups in LF, HF and LF/HF (Table 3).

**Table 3:** Comparison of DC, AC and HRV parameters between patients in the VT and non-VT groups

Variable	Non-VT group (*n* = 48)	VT group (*n* = 22)	*P*
DC (ms)	5.62 ± 1.75	4.13 ± 1.72	0.036
AC (ms)	-5.82 ± 1.84	-4.76 ± 1.69	0.040
SDNN (ms)	78.7 ± 22.1	68.2 ± 19.8	0.041
SDANN (ms)	68.7 ± 11.0	58.5 ± 10.5	0.043
rMSSD (ms)	21.3 ± 8.2	14.6 ± 6.2	0.045
LF (ms^2^)	1152.2 ± 440.5	1250.2 ± 408.6	0.068
HF (ms^2^)	221.5 ± 34.1	210.8 ± 27.6	0.071
LF/HF ratio	5.1 ± 2.0	5.9 ± 2.5	0.070

Data presented as the mean ± standard deviation

*AC* acceleration capacity of the heart rate, *DC* deceleration capacity of the heart rate, *HF* high-frequency power, *LF* low-frequency power, *rMSSD* the root mean square value of the standard deviations of the successive differences between adjacent normal RR intervals in the whole 24-h period, *SDANN* the standard deviation of the average normal RR intervals in all 5-min segments, *SDNN* the standard deviation of all normal RR intervals, *VT* ventricular tachycardia

### Correlations of DC and AC with HRV parameters in the VT group

In the VT group, DC was correlated positively with SDNN, SDANN, rMSSD and HF, and negatively with LF and LF/HF (*P* < 0.05; Table 4). AC was correlated negatively with SDNN, SDANN and rMSSD (*P* < 0.05), but was not correlated with LF, HF and LF/HF (Table 4).

**Table 4:** Correlations of DC and AC with HRV parameters in the VT group

Variable	DC		AC	
	*r*	*P*	*r*	*P*
SDNN (ms)	0.716	<0.001	-0.682	<0.001
SDANN (ms)	0.595	0.005	-0.567	0.006
rMSSD (ms)	0.571	0.007	-0.548	0.008
LF (ms^2^)	-0.518	0.038	0.413	0.057
HF (ms^2^)	0.486	0.043	-0.398	0.062
LF/HF ratio	-0.458	0.041	0.387	0.060

*AC* acceleration capacity of the heart rate, *DC* deceleration capacity of the heart rate, *HF* high-frequency power, *LF* low-frequency power, *rMSSD* the root mean square value of the standard deviations of the successive differences between adjacent normal RR intervals in the whole 24-h period, *SDANN* the standard deviation of the average normal RR intervals in all 5-min segments, *SDNN* the standard deviation of all normal RR intervals

## Discussion

The main findings of the present study were that, in Chinese patients with AECOPD, those with VT had a lower DC, SDNN, SDANN and rMSSD, and a higher AC, than those without VT. Furthermore, DC was correlated positively with SDNN, SDANN, rMSSD and HF, and negatively with LF and LF/HF, while AC was correlated negatively with SDNN, SDANN and rMSSD. These data indicate that patients with AECOPD and VT have greater impairment of cardiac autonomic function than patients without VT, suggesting that these patients may also be at increased risk of sudden death.

In patients with COPD, the effects of chronic airflow obstruction on the cardiovascular system and cardiac autonomic nerves manifest mainly as a limitation of mobility, cardiac autonomic dysfunction, increased resting heart rate and decreased BRS [7, 23]. Impaired autonomic function may also cause airway hyperreactivity, pulmonary vasoconstriction, aggravation of hypoxia, and progression of COPD [24]. Patients with AECOPD and hypoxemia may experience long-term atrial and ventricular hypoxia, leading to increased sympathetic nerve activity, decreased vagal nerve activity, impaired sinus node function, an acceleration of the heart rate, a shortening of the diastolic interval and hence reduced myocardial perfusion, increased myocardial oxygen consumption, increased levels of byproducts of local ischemia, a declined ventricular fibrillation threshold, increased occurrence of malignant arrhythmias and increased risk of sudden cardiac death [10, 11].

The results of the present study show that the VT group had a lower DC value and higher AC value than the non-VT group, suggesting that patients with AECOPD and VT suffer from enhanced sympathetic nerve activity, weakened vagal nerve activity and impaired autonomic nervous function, as compared to patients with AECOPD in the absence of VT. This would tend to induce electrophysiologic instability in the heart, which can lead to aggravation of pre-existing abnormal endocardial electric activity in patients with non-sustained VT, increasing the risks of lethal reentrant VT and sudden cardiac death [10, 11, 25]. Notably, 28.6 % of patients in the present study had non-sustained VT, higher than the value reported by Konecny et al. [6]. A possible explanation is that the present study enrolled only patients with AECOPD, in whom autonomic function impairment was likely more severe than that in patients without AECOPD.

Although AECOPD is thought to aggravate airflow obstruction, worsen respiratory symptoms and exacerbate ANS imbalance, the underlying mechanisms remain incompletely understood. One possibility is that airway obstruction during AECOPD leads to carbon dioxide retention and hypoxia, stimulating carotid sinus chemoreceptors and pulmonary stretch receptors to strengthen sympathetic nerve activity and reduce vagal tone [26–28]. Another possibility is that patients with AECOPD may have right atrial and ventricular enlargement, ventricular wall hypertrophy and atrophy, and interstitial edema. Necrotic lesions may have been replaced by fibrous tissue, leading to a decrease in the number of vagal receptors, and thus increased sympathetic nerve activity. A third potential mechanism is that, normally, the right sympathetic and vagal nerves dominate the sinus node, with sympathetic nerves distributed along the epicardium, and vagal nerves distributed along the endocardium. Long-term hypoxia-ischemia in the right atrium and ventricle will stimulate baroreceptors and chemoreceptors in the atrioventricular wall, mainly damaging the vagal nerve fibers and leading to a decreased DC.

HRV can be used to assess sympathetic and vagal tone and balance, and their impact on cardiovascular activity. A decreased HRV was suggestive of impaired cardiac autonomic function, and was associated with increased risk of sudden death [29, 30]. The present study showed that SDNN, SDANN, rMSSD and HF were lower in the VT group than in the non-VT group, indicating significant impairment of cardiac autonomic function; this is consistent with previously published results [31, 32]. In the VT group, DC correlated positively with SDNN, SDANN, rMSSD and HF, and negatively with LF and LF/HF, while AC correlated negatively with SDNN, SDANN and rMSSD. This suggests that DC and AC correlate well with more traditional indicators of autonomic function.

Under normal conditions, heart rate regulation by ANS is subtle and rapid, such that changes can occur from one cardiac cycle to the next. This is reflected in each cardiac cycle, and these regulatory ‘signatures’ of the autonomic nerves can be extracted as DC and AC using PRSA technology. DC and AC provide separate quantitative analyses of the effects of vagal and sympathetic nerves system, with pathologic changes in DC thought to be of greater clinical significance than changes in AC [14]. In the 24-h ECG, a prolonged cardiac cycle compared with the preceding one is indicative of heart rate deceleration brought about by the vagal nerve. Therefore, DC can be used to assess vagal activity, and could potentially be used in screening to identify patients at high risk of sudden death. Furthermore, the techniques required for determination of DC and AC are straightforward to use and do not require imposition of specific conditions or additional interventions such as administration of drugs. Thus, PRSA technology is capable of quantitatively assessing the effects of vagal and sympathetic nerves with high reliability, sensitivity and specificity, and has advantages over alternative commonly used approaches [13].

We studied cardiac autonomic function in patients with AECOPD, and found that DC is decreased and AC increased in patients with AECOPD and VT. However, there are several limitations to our study. First, this was a retrospective study, hence selection and reporting bias cannot be excluded. Second, the sample size was small, so the study may have been underpowered to detect real differences for some comparisons. Third, this was a single-center study, and the cohort may not be representative of the general patient population in China or elsewhere. Fourth, comparator groups (e.g. age-matched patients with COPD but without an acute exacerbation, and age-matched healthy controls) were not included, precluding the determination of whether autonomic dysfunction in patients with AECOPD is greater than that in patients with COPD not experiencing acute exacerbation or healthy individuals. Fifth, patients taking drugs that might affect ANS function and atrial fibrillation/flutter were excluded from the study, which would have resulted in a degree of bias in patient selection. Sixth, some patients in the VT group were receiving β2-agonists, anticholinergics, hormones; these drugs may have influenced the parameters measured using PRSA. Seventh, It was reported that amiodarone did not decrease time domain measures of HRV in patients with chronic ventricular arrhythmia [33]. There are no data available regarding the influence of amiodarone on the PRSA-derived cardiac indices, but it is possible that it would be a potential confounder on the autonomic nervous system and thus on the measured parameters. Therefore, multi-center, prospective studies with a larger sample size are merited to confirm and extend our findings.

## Conclusions

The present study determined that DC is reduced in patients with AECOPD accompanied by VT, reflecting an imbalance in cardiac autonomic regulation that, potentially, could increase the risk of sudden death. This suggests that clinical intervention in patients with AECOPD and VT should not only focus on actively improving ventilation, reducing inflammation and suppressing arrhythmia, but also target ANS dysfunction to maintain myocardial electrical stability, elevate the ventricular fibrillation threshold, and reduce the risk of sudden death.

## Abbreviations

COPD: Chronic obstructive pulmonary disease
PRSA: Phase-rectified signal averaging
AC: Acceleration capacity of heart rate
DC: Deceleration capacity of heart rate
AECOPD: Acute exacerbation of COPD
VT: Ventricular tachycardia
SDNN: The standard deviation of normal sinus RR intervals
SDANN: The standard deviation of average RR intervals in 5-min segments
rMSSD: The root mean square of standard deviations of differences between adjacent normal RR intervals
LF: Low-frequency power
HF: High-frequency power
ANS: Autonomic nervous system
HRV: Heart rate variability
BRS: Baroreflex sensitivity
HRT: Heart rate turbulence
ECG: Electrocardiogram
BUN: Blood urea nitrogen
Cr: Serum creatinine
FBG: Fasting blood glucose
TC: Serum total cholesterol
LVEF: Left ventricular ejection fraction
mMRC: Modified British Medical Research Council
